# Design principles for effective video-based professional development

**DOI:** 10.1186/s40594-017-0091-2

**Published:** 2017-11-24

**Authors:** Kathleen J. Roth, Jody Bintz, Nicole I. Z. Wickler, Connie Hvidsten, Joseph Taylor, Paul M. Beardsley, Arlo Caine, Christopher D. Wilson

**Affiliations:** 1 California State Polytechnic University, Pomona, Building 4-2-515, 3801 West Temple Ave, Pomona, CA 91768 USA; 2 Biological Sciences Curriculum Study (BSCS), 5415 Mark Dabling Blvd., Colorado Springs, CO 80918 USA

**Keywords:** Professional development, Video-based professional development, Design principles, Facilitation of professional development, Teacher leaders, Science teaching and learning, Elementary science, Experimental study, Sustainability

## Abstract

**Background:**

Most studies of teacher professional development (PD) do not rigorously test impact on teaching practice and student learning. This makes it difficult to define what is truly “effective.” The *Science Teachers Learning from Lesson Analysis* (STeLLA) PD program, in contrast, was studied in a cluster randomized experimental design that examined impact on teaching practice and student learning. The STeLLA video-based PD (VbPD) program demonstrated significant impact, with high effect sizes, on elementary teachers’ science teaching practice and their students’ learning. Previously published reports provide details about research methods and findings but only broad sketches of the STeLLA program design and implementation. Deeper explorations of the STeLLA design principles can contribute evidence-based knowledge about the features of effective PD and enrich the existing but limited consensus model of effective PD. This article addresses the following questions:

- What design principles guided the development, implementation, leadership, and scaling up of a video-based PD program that had significant impact on student learning?
- What do the STeLLA design principles contribute to the existing knowledge base about effective video-based PD?

**Results:**

Results from rigorous studies of the STeLLA program are summarized in this paper; details are reported elsewhere and included here as supplementary materials. This article is not a standard research results paper but instead describes the design principles guiding the development, implementation, leadership, and scaling up of the STeLLA VbPD program.

**Conclusions:**

The authors argue that this set of design principles is powerful for four reasons: 1) its demonstrated impact on teaching practice and student learning, 2) its strong theoretical and research foundations, 3) the stability and usefulness of the design principles as implemented in changing contexts over a 10-year period, and 4) the coherence and interconnectedness of the principles. The STeLLA VbPD design principles contribute to the field by empirically supporting and advancing the existing consensus model of effective PD. Further study can build on this effort to strengthen our understanding of effective PD based on evidence of impact on teaching practice and student learning.

What are the key features of effective professional development? Researchers have been writing about this question for decades (Ball and Cohen 1999; Desimone, Porter, Garet, Yoon, and Birman 2002; Garet, Porter, Desimone, Birman, and Yoon 2001; Kennedy 1999; Loucks-Horsley, Hewson, Love, and Stiles 1998; National Academy of Sciences, Engineering, and Medicine (NASEM) 2015; Wilson and Berne 1999; Wilson 2013). But answers to this question have not changed much over the years because most studies of teacher professional development (PD)—today, as in the past—do not include rigorous tests of impact on teaching practice and student learning and do not include strong comparisons to other PD approaches (Borko 2004; NASEM 2015; Wilson 2013). This makes it difficult to define what is truly “effective” teacher professional development.

In the USA, the need for such knowledge has been made more urgent by the release of the *Next Generation Science Standards* (NGSS Lead States 2013) which sets expectations for science teaching and learning that are in striking contrast with current US science teaching practice (Banilower, Smith, Pasley, and Weiss 2006; Roth et al., 2006; Weiss, Pasley, Smith, Banilower, and Heck, D.J. 2003). This has resulted in a high demand for professional development opportunities that support teachers in changing their science teaching practice to better engage students in using science practices—such as explanation and argumentation—to develop rich understandings of science ideas and concepts. Science educators in other countries face similar challenges. But developers and leaders of science professional development efforts are hampered in designing and implementing such PD opportunities by the dearth of research that follows the pathway of impact of PD programs on teacher knowledge, teaching practice, and student learning in rigorous comparison with other PD approaches.

The Science Teachers Learning from Lesson Analysis (STeLLA) PD program is one of a handful of programs that has been studied in a cluster randomized experimental study where the program was compared with another PD program of equal duration and where analysis included impact on teaching practice and student learning. This yearlong, video-based, analysis-of-practice PD program demonstrated significant impact, with high effect sizes, on upper elementary teachers’ science content knowledge, teachers’ pedagogical content knowledge (PCK), teachers’ science teaching practice, and, most importantly, on student learning (Fig. 1). As a result of strong findings from this and other studies of the program, the scalability and sustainability of this program when led by K-6 teacher leaders in a high-needs, urban school district is currently being tested in a project titled, *Reinvigorating Elementary Science through a Partnership with California Teachers* (RESPeCT).

**Fig. 1:**
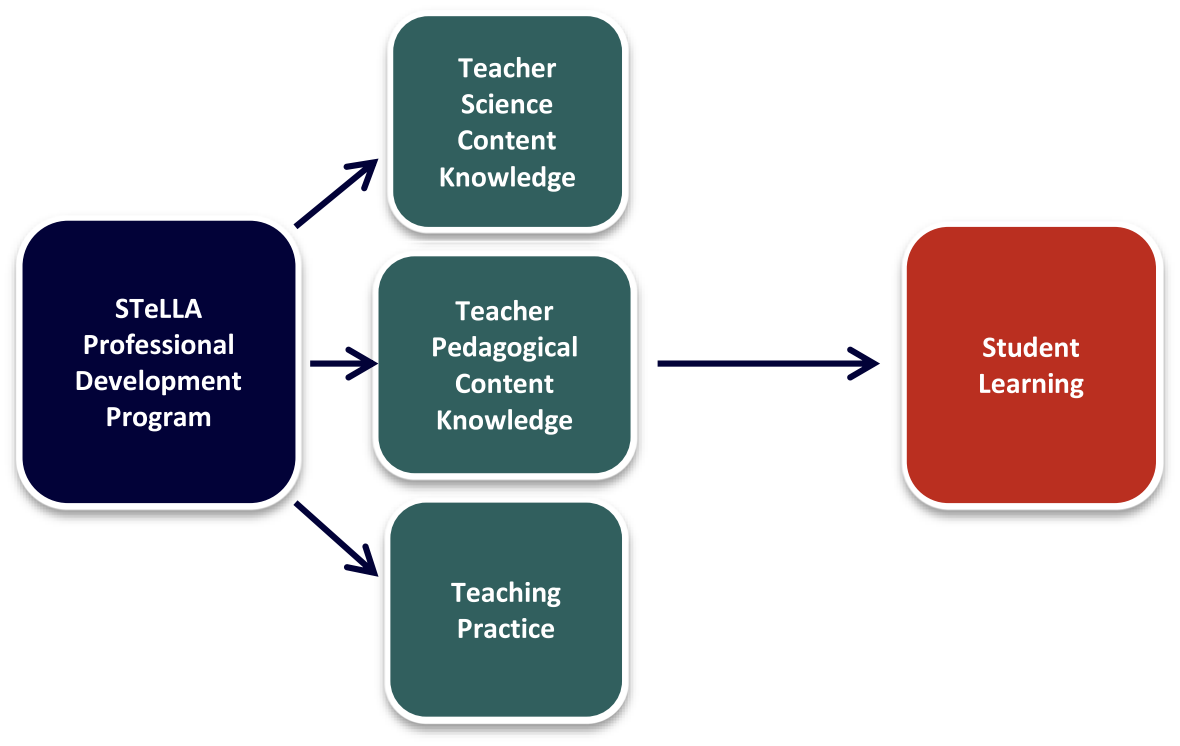
STELLA professional development program hypothesized pathway of influence

Research reports about this STeLLA line of research provide details about research methods that explain how we know that the STeLLA video-based, analysis-of-practice program is effective, but they provide only a surface-level sketch of the program design and implementation (Roth, Garnier, Chen, Lemmens, Schwille, & Wickler, 2011; Taylor, Roth, Wilson, Stuhlsatz, & Tipton, 2016). These previously published research reports provide strong evidence that video analysis is a powerful approach to science teacher PD, but they fall short of helping the field understand what it takes to design, implement, and scale such a video-based PD (VbPD) program. To address this gap, this article goes “behind the scenes” to examine the design principles undergirding the development, implementation, leadership, and scaling up of this effective PD program. While the research results will be briefly summarized, this article is not a standard research results paper. Instead, it explores the following questions:

- What design principles guided the development, implementation, leadership, and scaling up of a video-based professional development program that had significant impacts on teaching practice and student learning?
- What do the STeLLA design principles contribute to the existing knowledge base about effective video-based, analysis-of-practice PD?

To answer the first question, we describe in the body of the paper how the STeLLA program’s design principles shaped (a) the substance and form of the STeLLA approach to video-based analysis of practice, (b) the implementation of video analysis in the STeLLA program, (c) the leadership of the program, and (d) the scaling of this VbPD program through the development of teacher PD leaders in a school district-university partnership. To address the second question, we begin the paper by placing this work in the context of the larger body of research on teacher professional development, focusing on the limitations of the existing “consensus model of effective PD” and the need to better understand what it takes for PD to make a difference in terms of student learning. In the discussion at the end of the paper, we consider the power of the STeLLA approach to video-based analysis of practice and ways in which the STeLLA design principles advance and deepen the field’s current understanding of the consensus model of effective PD. In the end, we hope readers will find their understanding of the STeLLA VbPD design principles to be useful in their own work in designing, implementing, sustaining, and rigorously studying the impact of VbPD approaches. In this way, the science education community can move forward toward a more refined consensus model of PD that is supported by research showing changes in teaching practice that are linked to improved student learning.

## The problem: the limitations of the consensus model of effective PD

While there is widespread agreement that teacher professional development is key to improving teaching and learning, there is little research that examines the impact of various PD approaches on student learning—in science or in other subject matter areas. Despite this lack of student learning evidence, there is a widely accepted consensus model of effective professional development (Desimone 2009; Wilson 2013). In this consensus model, effective PD:

(i) Focuses on specific subject matter content,
(ii) Engages teachers in active learning,
(iii) Is consistent with reform documents and school, district, and state policies and practice,
(iv) Is of sufficient duration (both in intensity and contact hours), and
(v) Involves the collective participation of teachers (e.g., at a school, at a grade level).

This consensus model was generated from studies whose research designs are weakened by heavy reliance on teacher self-report data, small sample sizes, and lack of control or comparison groups (Desimone 2009: Wilson 2013; Yoon, Duncan, Lee, Scarloss, and Shapley 2007; NASEM 2015). For example, studies by Garet et al. (2001) and Desimone et al. (2002) relied on survey responses from science and math teachers who had participated in federally funded Eisenhower PD programs. In addition, most PD studies focus primarily on teacher knowledge and belief outcomes rather than on teaching practice and student learning (Little 2011; Desimone 2009; Yoon et al. 2007; NASEM 2015). In a review of over 1300 studies of professional development that examined the relationship between PD and student learning, only nine had randomized controlled or quasi-experimental designs that included measures of both teacher and student outcomes and only two of these studies examined science PD (Yoon et al. 2007).

Over the past two decades, various authors have made conceptual arguments to characterize this consensus view and have written syntheses of the existing research on PD (across subject matters) (Ball and Cohen 1999; Blank, de las Alas, and Smith 2008; Borko 2004; Borko, Jacobs, and Koellner 2010; Darling-Hammond, Chung Wei, Andree, Richardson, and Orphanos 2009; Hawley and Valli 2007; NASEM 2015; Wilson and Berne 1999; Wilson 2013; Yoon et al. 2007). The most recent reviews of PD for STEM educators sound strikingly similar to those of reports from a decade ago: There is consensus about what high-quality PD should look like and little empirical evidence to support that consensus model (Wilson 2013; NASEM 2015).

In fact, some research studies challenge the consensus model. Studies that test separately for each feature of the model reveal inconsistent results in terms of impact (Garet et al. 2008, 2011; Scher and O’Reilly 2009). And when tested using rigorous experimental designs that examine impact on student learning, the consensus model is not always predictive of student learning (Garet 2016; Heller, Daehler, Wong, Shinohara, and Miratrix 2012; National Center for Education Evaluation and Regional Assistance (NCEE) 2017). These results suggest that the consensus model is not sufficient for describing PD that is effective in terms of student learning. Instead, the consensus model is perhaps capturing “surface characteristics and not the mechanisms that account for teacher [and student] learning” (NASEM 2015, p. 118).

Despite the limitations of the research supporting the consensus model, it has been widely used as a guide in designing professional development programs—in science and in other subject areas. In their reviews of research on science professional development, for example, Capps, Crawford, and Constas (2012) and van Driel and Berry (2012) found that most of the PD programs that were reviewed reflected the consensus model. This is also true of more recent studies of science professional development that examined both teacher and student learning outcomes (Greenleaf et al. 2011; Heller et al. 2012; Johnson and Fargo 2010; Penuel, Gallagher, and Moorthy 2011; Roth et al., 2011; Taylor et al., 2016).

So, there is wide use of the consensus model despite a lack of strong evidence to support its usefulness as a predictor of student learning. There is also much interest in the use of video as a strategy for supporting teachers in the active learning called for in the consensus model. The STeLLA PD program uses a video-based approach and has been demonstrated to have impact on student learning (the research findings will be summarized in the next section). What can our experiences from this program contribute to the field that goes beyond the features identified in the consensus model of effective professional development? Since the STeLLA program demonstrated evidence of student learning, this is a good place to dig deeper—to go beyond the surface, to examine: What is going on in the STeLLA approach to video-based PD that makes it have such dramatic impact?

In this article, we share the STeLLA design principles and consider ways that they might be a starting place for developing a more specific and useful model of effective VbPD. Before describing the design principles, we provide a brief summary of the research that supports our assertions of the impact of the STeLLA program.

## Research background: the impact of the STeLLA VbPD program

Research documenting the impact of the STeLLA video-based professional development program on upper elementary teacher and student learning has been completed in two studies, which we will refer to as STeLLA-I and STeLLA-II. Ongoing research is extending this line of research by examining impact on K-6 teachers and their students when university science faculty and later teacher leaders in an urban district facilitate the program in the *Reinvigorating Elementary Science through a Partnership with California Teachers* (RESPeCT) program. Additional studies are currently studying impact of the STeLLA VbPD approach on preservice/first year elementary teachers and their students and on high school teachers and their students. In this section, we summarize findings from the two completed studies (STeLLA-I and STeLLA-II) and provide preliminary findings from the RESPeCT study which is testing the scalability and sustainability of this program. Details of the STeLLA-I and STeLLA-II studies can be found in previously published reports which are available in the supplementary materials (Roth et al., 2011; Taylor et al., 2016).

In the original STeLLA-I study, the program was designed, piloted, and then studied using a quasi-experimental design. Forty-eight upper elementary teachers who were teaching in urban settings in Southern California volunteered for either the year-long STeLLA lesson analysis VbPD program or a 2-week summer science content deepening program. The content deepening program was an existing program (e.g., business as usual) based at the California State Polytechnic University at Pomona (CPP) which was supported by the California Subject Matter Projects network. The goal of this network was to engage university faculty and other educators in providing high-quality, content-specific professional development for teachers across the state of California in nine subject matter areas. The CPP math and science content deepening programs were popular with regional elementary teachers and had been in existence for years.

The STeLLA-I study examined teachers’ and students’ science content knowledge via written tests (pre-mid-post), teachers’ ability to use PCK in their analysis of video clips of science teaching (pre-mid-post), and teachers’ science teaching practice as captured in lesson videos (pre-post). Research findings (Roth et al., 2011) showed that, in comparison with teachers who received science content deepening PD only (*n* = 16), teachers experiencing the 1-year STeLLA VbPD lesson analysis program (*n* = 32) developed deeper science content knowledge (*p* < .001) and stronger abilities to use PCK to analyze science-teaching practice (*p* < .001). In addition, teachers in the STeLLA VbPD program increased their use of teaching strategies that made student thinking visible and contributed to the coherence of the science lesson (*p* < .01). Most importantly, their students’ learning showed significant improvement (*p* < .01, average effect size *d* = 0.47).

Led by the Biological Sciences Curriculum Study (BSCS), the STeLLA-II study tested the efficacy of the VbPD program using a randomized design (assignment to treatment group at the school level) to examine whether the program, when delivered in a new geographic area (urban, suburban, and rural schools along Colorado’s Front Range) and by PD leaders outside the original program development team, is as effective as a science content deepening program of equal duration. The content deepening program used in STeLLA-I was extended to become a program that supported teachers during the school year in addition to the summer institute. The strength of the comparison group is supported by teachers’ initial interest in being assigned to this program, teachers’ persistence when assigned to this program, and teachers’ feedback about the high quality of this experience in surveys and focus groups conducted by the external evaluation team. The experimental sample included 77 schools, 137 teachers, and 2823 students.

Analyses showed that the STeLLA video-based, analysis-of-practice intervention was more effective for both students and teachers than the content deepening program of equal duration (see Table 1) (Wilson, Taylor, Roth, Stuhlsatz, & Hvidsten, 2016). All effects on teachers were statistically significant with associated effect sizes ranging from 0.66 to 2.05. The effect of STeLLA on students was also statistically significant with an effect size of 0.68 which far exceeds empirical benchmarks for interventions for elementary school students (see Hill, Bloom, Black, and Lipsey 2008). While students from both groups showed significant growth in their science understanding, students whose teachers were in the STeLLA treatment group outperformed students from the content deepening group. Looking more closely at the results of student content tests, a distinction emerges between the types of questions the average students of STeLLA teachers were able to answer and those that the average student in the content deepening group could answer. Students of STeLLA teachers were better able to answer questions involving more scientific reasoning and application of science concepts in new contexts.

**Table 1:** STeLLA-II impact estimates

Outcome	Unstandardized treatment effect	Standard error	*t*	df	*p*	Hedges’ *g* (WWC)
Student achievement	6.11 [4.46, 7.76]	0.84	7.27	74	<.001	0.68 [0.60, 0.76]
Teacher content knowledge	4.77 [3.26, 6.28]	0.77	6.16	75	<.001	0.66 [0.31, 1.00]
Teacher pedagogical content knowledge	5.33 [3.74, 6.92]	0.81	6.58	75	<.001	1.17 [0.81, 1.53]
Teaching practice	15.60 [13.01, 18.19]	1.32	11.78	75	<.001	2.05 [1.63, 2.47]

At the teacher level, teachers in the STeLLA treatment group significantly outperformed teachers in the content deepening group on the science content knowledge assessment, despite the fact that they spent much less time focused on content deepening work. The effects of STeLLA on teachers’ pedagogical content knowledge and classroom practice were even larger. Mediation analyses suggested that only the teaching practice outcome had a statistically significant relationship with students’ science achievement scores (at the 5% significance level).

A major challenge for school districts and the science education research community is how to scale up rigorously tested video-based professional development experiences, such as STeLLA, so that they can reach larger numbers of teachers (and their students) in a manner that is practical and sustainable. The RESPeCT project is an NSF-funded partnership project that is studying whether the STeLLA VbPD approach can be scaled to eventually reach most K-6 teachers in an urban, high-needs, Title I school district. The partners are California State Polytechnic University at Pomona, the Pomona Unified School District (PUSD), and the nonprofit science education research organization, Biological Sciences Curriculum Study (BSCS). The goal is that by the end of the 5-year project, PUSD will have the capacity to continue to implement the STeLLA VbPD program with limited input from CPP and have a direct, lasting, and positive impact on their students’ learning. The key to this scale-up effort is the development of a cadre of K-6 grade level teacher leaders within the district.

The RESPeCT project is using a quasi-experimental research design with a comparison group of “business-as-usual” teachers from schools matched to treatment school demographics. The research phase of the project is still underway but preliminary analyses of teachers' science content learning and students' science learning in grades 1–6 (kindergarten assessments are still being analyzed) demonstrate significant growth in all content areas as compared with teacher and student learning in business-as-usual classrooms. For example, initial analyses of student content learning gains show a mean effect size of 0.97 across 11 content modules (one module in grade 1, two modules for each grades 2–6; range 0.32–1.86). For teacher content knowledge, there is a strong interaction between the pre- and posttest and group assignment (treatment versus comparison). The null hypothesis that gains by STeLLA treatment teachers and comparison teachers are equal can be rejected with a high degree of confidence (*p* < .0001). An effect size of 1.6 shows that the treatment teachers made very large gains in their content knowledge versus comparison teachers.

## Overview of STeLLA VbPD design principles

What mak﻿es this VbPD program so effective? Although the STeLLA VbPD program includes the surface features of the consensus model, we assert that these features do not explain the program’s power. It is not just that teachers were engaged in content-specific, collaborative, video-based analysis-of-practice. Instead, the program has been guided by a more specific set of 19 design principles that challenge us to dig beneath the surface of the consensus model (see Table 2). In the next sections, we describe how these design principles guided the substance and form, implementation, leadership, and scaling up of the STeLLA VbPD program.

**Table 2:** Design principles in the STeLLA line of research

Foundational design principles	
1. *Conceptual framework*: A conceptual framework that is grounded in research about effective science teaching and learning and effective professional development defines the core substance of the program.	
2. *Specified teacher and student learning goals*: The program is guided by clearly specified teacher and student learning goals that are closely tied to the conceptual framework—science content, pedagogical content knowledge, teaching practice.	
3. *Program substance prioritizes depth over breadth*: The substance of the program is limited in scope to a few key science ideas in two topic areas, the STeLLA teaching strategies, and an analysis process that focuses on the Student Thinking and Science Content Storyline Lenses.	
4. *Theory of teacher learning*: A situated cognition theory of teacher learning and a cognitive apprenticeship instructional theory guides the design and sequencing of teacher learning experiences.	
Program learning experiences	
5. *Video-based analysis of practice*: Analysis of classroom teaching and learning using classroom video and student work is a core teacher learning activity.	
6. *Science content learning experiences*: Teachers’ science content learning is closely linked to analysis-of-practice work. Science content learning experiences that emerge from lesson video analysis are prioritized.	
7. *Scaffolded teaching practice*: Teachers have scaffolded opportunities to practice using the science and the teaching strategies they are learning about (STeLLA curriculum materials).	
Program form	
8. *Duration and intensity*: The program is of significant duration (2 weeks summer and one academic year) and intensity to make significant changes in teacher knowledge and practice.	
9. *Collaborative learning*: The development of small, face-to-face study group learning communities in which grade-level teachers share their practice enables deeper analysis of practice.	
Program resources	
10. *Shared science content and curriculum*: Content-specific and curriculum-specific analysis of practice provides shared experiences that allow for deeper analysis of practice and development of common understandings of the content and the teaching strategies.	
11. *Educative lesson plans and assessments*: Grade-level and content-specific educative curriculum materials support the use of STeLLA strategies, provide anticipated student responses to questions and activities, and highlight how the science content storyline is developing.	
12. *Both exemplar and participant videos*: Exemplar classroom videocases from teachers outside the study are used to develop an initial understanding of the strategies and the analysis process. Later in the program video analysis focuses on videos from participants’ classrooms.	
13. *Analysis tools and processes*: Analysis tools and processes scaffold teacher learning from analysis.	
14. *Reference materials*: Teachers make regular use of STeLLA reference materials to assure shared and grounded understandings.	
Program leadership	
15. *PD leadership*: PD leadership/facilitation plays a critical role in deepening teacher learning from analysis of practice.	
16. *PD leader knowledge and decision-making abilities*: PD leadership/facilitation requires the ability to draw from a rich base of knowledge (Pedagogical Content Knowledge for PD ﻿Leaders) to make planning and in-the-moment, program-aligned decisions and adaptations.	
Scalability and sustainability	
17. *PD leader development*: PD leaders are continuously supported in learning how to effectively implement this program.	
18. *Partnership development*: A partnership of science education experts and researchers, scientists and mathematicians, and school personnel at multiple levels are involved in ensuring successful implementation across a school district.	
19. *Scalability and sustainability*: The program is modified in ways that preserve the above design features while incorporating features that will contribute to the program’s sustainability and scalability.	

## Foundational design principles of the STeLLA video-based PD program

We begin with four foundational design principles that have played central roles throughout the development, implementation, leadership, and scaling up of the program.

### Design principle 1: conceptual framework

The STeLLA VbPD program was designed to improve elementary science teaching at a time when science teaching and science PD activities were low on teachers’ priority lists (Dorph, Shields, Tiffany-Morales, Hartry, and McCaffrey 2011). At the same time, researchers and PD providers were advocating that effective PD engages teachers in sustained, collaborative analysis of practice over time using artifacts of practice such as videos and student work (Ball and Cohen 1999; Garet et al. 2001). How could we interest elementary teachers in participating in such sustained science PD? In this context, the STeLLA research and design team decided to explore what would be possible if elementary teachers engaged in video-based, analysis-of-practice PD activities intensively for 1 year. This limited time frame raised important questions about the substance of the PD program: What is possible within a 1-year period? Is it possible to change science teaching practice enough in just 1 year to impact student learning? To achieve such a goal within a 1-year period, it was assumed that the substance of the program would need to be tightly focused on a few ideas and teaching practices addressed in depth and over time.

**Fig. 2:**
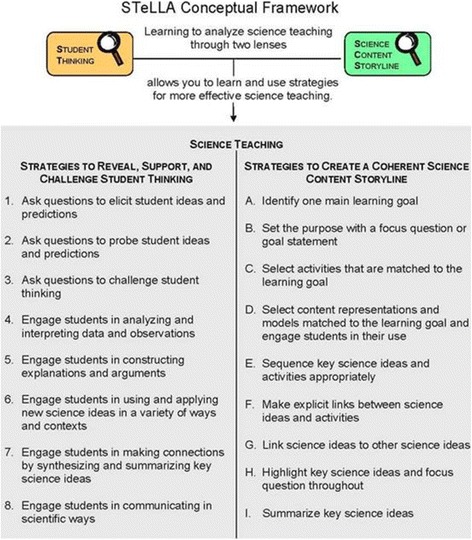
STeLLA conceptual framework

Based on a thorough review of the research on effective PD and the much larger research base about effective science teaching, the STeLLA-I design team made difficult choices about what to focus on and what to leave out. The result was a conceptual framework for the entire PD program (see Fig. 2). The framework centers on two types of pedagogical content knowledge (PCK) that were hypothesized to have the most impact on teacher learning, science teaching practice, and student learning within a 1-year time frame: (1) revealing, supporting, and challenging students’ ways of thinking about specific science content, and (2) supporting students in constructing coherent science content storylines. Note that we did not attempt to address all kinds of pedagogical content knowledge. We selected these two dimensions for three reasons. First, they represent an important subset of the topic-specific professional knowledge defined in expert models of PCK (Gess-Newsome 2015). Second, we hypothesized that these two dimensions would be impacted by the video analysis intervention. And finally, these are perspectives that teachers do not typically use to frame and guide their planning and teaching (Sherin and Van Es 2002; Zannoni and Santagata 2002). To focus on these aspects of PCK, teachers in the program use two lenses to guide their video-based analysis work and their classroom science teaching practice: the Student Thinking Lens and the Science Content Storyline Lens.

The Student Thinking Lens focuses teachers’ attention on revealing, supporting, and challenging student thinking. It builds on a large body of research regarding students’ ideas about natural phenomena, how student ideas might influence science teaching, and the importance of attending to student ideas and cultures in supporting science learning of students from groups underrepresented in science (National Research Council (NRC) 2000; NRC 2005; NRC 2007). Eight teaching strategies support teachers in enacting the Student Thinking Lens in their classrooms (see definitions in Appendix 1). These teaching strategies reflect an instructional model that:

- Elicits students’ initial ideas and predictions about phenomena,
- Engages students with data, observations, and models that challenge their initial thinking and reasoning,
- Supports students in constructing explanations from evidence,
- Challenges students to use and apply new ideas to explain new phenomena, and
- Focuses students on making connections by synthesizing and summarizing key ideas.

Throughout all of this, the teacher probes student thinking to find out how students are making sense of new data or ideas, challenges students to stretch their thinking and to make new connections, and teaches students to communicate in scientific ways (such as making claims, providing evidence and reasoning to support claims, and listening and responding to others’ ideas).

The Science Content Storyline Lens focuses attention on how the science ideas in a science lesson or unit are sequenced and linked to one another and to lesson activities to help students construct a coherent “story” that makes sense to them. The Trends in International Mathematics and Science Study’s (TIMSS) video analysis of science teaching and Horizon’s *Looking Inside the Classroom* observational study of science teaching in the USA both identified that US science lessons frequently fail to support students in making connections between science classroom activities and the development of science ideas and explanations (Roth et al., 2006; Weiss et al. 2003). The Science Content Storyline Lens addresses this gap by supporting teachers in using nine planning and teaching strategies that help students build coherent content storylines (see definitions in Appendix 2)

Grounded in research, the conceptual framework serves as the foundation and the centerpiece of the PD program, with all summer and school-year sessions organized around the STeLLA lenses and strategies. It anchors the program and assures program coherence by keeping the substance and activities in the program tightly focused on two analytical lenses and a few key ideas and teaching strategies that can be explored in depth and developed into teachers’ practice.

### Design principle 2: specified teacher and student learning goals

The STeLLA VbPD program is guided by clearly specified teacher and student learning goals that are closely tied to the conceptual framework. Identifying and articulating these learning goals from the beginning is essential for designing and implementing both science lessons and PD sessions that are coherent, focused, and supportive of student/teacher learning. In our work, learning goals for students focus on understanding and using core science ideas. Learning goals for teachers include science content knowledge, pedagogical content knowledge related to classroom science teaching, and abilities to use both these types of knowledge to analyze and teach science.

#### Science content learning goals for students and teachers

The goal in STeLLA work is for teachers and students to develop understandings of key science ideas and related crosscutting concepts through the use of science practices such as analyzing and interpreting data and constructing explanations (STeLLA strategies 4 and 5, see Fig. 2). This aligns with the emphasis in the NGSS that school science learning should parallel how scientists use science practices to develop conceptual understandings of the world around us (NGSS Lead States 2013). By developing understandings of key science ideas through such evidence-based reasoning, we expect students and teachers to be able to use and apply these ideas to analyze, interpret, and/or explain new data or phenomena that they encounter (STeLLA strategy 6, see Fig. 2). For this reason, our assessments include reasoning items which challenge students and teachers to use their understandings of core science ideas to analyze or explain data or representations that are new to them.

The STeLLA conceptual learning goals are stated as complete sentence ideas (similar to what is written as Disciplinary Core Ideas and Crosscutting Concepts in the NGSS). For each grade level in STeLLA-I and STeLLA-II, we identified student and teacher sets of science learning goals in two content areas that were in the state standards and school curricula of participating teachers. In the RESPeCT project, which started after the release of the NGSS, these content learning goals focused largely on NGSS-defined disciplinary core ideas and crosscutting concepts.

Several criteria were used to select the science learning goals. Priority was given to ideas that are (a) challenging for teachers and students, (b) that can be used to explain a variety of phenomena in teachers’ and students’ experience, and (c) that are supported by a research base that identifies common student difficulties and naïve theories related to learning this content. In addition, we wanted the subset of approximately 5–10 science ideas in each content area to be interconnected so that they could all be used together in culminating “use and apply” problem solving tasks. All lesson plans, content deepening work, and video analysis work remained focused on this set of interconnected ideas.

The *teacher* science content learning goals include the student science learning goals but go beyond them so that teachers have a deeper understanding of the science ideas they will be teaching and a better sense of the future trajectory of their students’ learning in this content area. The teacher science learning goals are addressed in the program in two main contexts: (a) through content deepening activities for teachers in the summer institute that are similar to those in the lesson plans for students, and (b) through opportunities that arise in the context of lesson video analysis throughout the program.

#### Additional learning goals for teachers

In addition to science content learning goals, STeLLA teachers are also expected to develop pedagogical content knowledge related to the Student Thinking and Science Content Storyline Lenses and teaching strategies. Furthermore, it is a goal for teachers to be able to use their content knowledge and their pedagogical content knowledge to analyze science teaching and learning. A final broad learning goal is for teachers to use their content and pedagogical content knowledge about the STeLLA lenses and strategies in their science teaching practice. The conceptual framework outlines the specific teaching strategies that teachers learn to understand and use (Fig. 2).

**Fig. 3:**
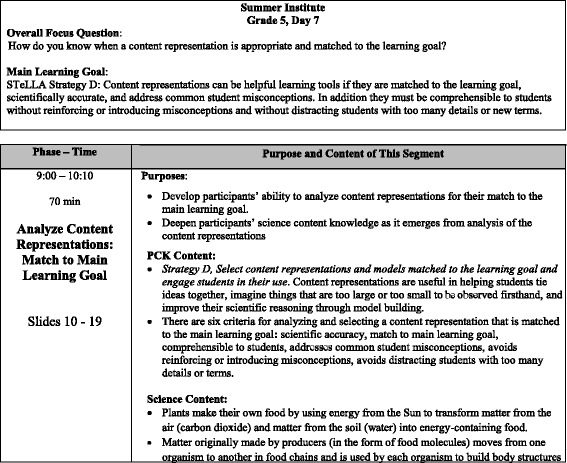
Summer institute day 7, grade 5 teacher learning goals

These broad teacher learning goals related to PCK, analysis abilities, and teaching practice were useful in creating the overall design of the STeLLA program and the development of research assessments. However, these overall program goals were insufficient for mapping out the daily PD sessions during the summer institutes and school year study group sessions. Over time, especially as we began to develop new PD leaders and to write detailed PD leader guides, we recognized the need to state more specifically the teacher learning goals for each professional development session and for each segment of a PD session. We recognized that just as students’ science lessons are more coherent and powerful if they remain clearly focused on one main learning goal throughout a lesson (STeLLA strategy A, see Fig. 2), so our PD sessions are more coherent and powerful if we remain focused on what we expect teacher participants to learn from a particular session and from particular segments of a PD session. Figure 3 shows how the STeLLA PD leader guide provides fifth grade PD leaders with specific statements of purpose and intended learning goals for a 70-min time segment that occurs during a full-day summer institute session.

In STeLLA VbPD, such specificity of the learning goals is critical in assuring coherence among all the components of the program. It plays a key role in developing the science lesson plans and videocases, in selecting video clips to use for analysis, and in deciding how to focus the video analysis discussions. Video analysis work is time intensive, so planning for a productive session is important. Video clips for STeLLA analysis are selected not just because they are interesting but because they will help teachers wrestle productively with intended learning goals—both specific science content learning goals and pedagogical learning goals.

### Design principle 3: program substance prioritizes depth versus breadth

A key design feature of all STeLLA-related programs is depth over breadth—maintaining a relatively small set of teacher learning goals that are introduced early in the program and then revisited and practiced throughout the program. The substance of the program is limited to a few core science ideas in two topic areas, the STeLLA teaching strategies, and the STeLLA video analysis process that uses the Student Thinking and Science Content Storyline Lenses as analytical tools. In parallel with what we know about students’ needs in developing understandings of science ideas, teachers also need multiple opportunities to use and analyze the science content ideas and the STeLLA teaching strategies that they are learning. Teachers initially encounter ﻿al﻿most﻿ all of the program’s science content ideas and STeLLA teaching strategies during the summer institute. Across the school year, only a few new learning goals are introduced. Instead, teachers have multiple opportunities to use and deepen their understandings of previously introduced ideas in their teaching and in their collaborative analyses of videos and student work.

### Design principle 4: theory of teacher learning

Many PD programs are not guided by an articulated theory of teacher learning (Ball and Cohen 1999; Borko 2004; Putnam and Borko 2000). In the STeLLA program, in contrast, decisions about program design are explicitly guided by a situated cognition theory of teacher learning and a cognitive apprenticeship model of instruction. Situated cognition posits that learning is naturally tied to authentic activity, context, and culture (Collins 2006; Lave and Wenger 1991). In line with this theoretical stance, the STeLLA program is organized to support grade-level specific, classroom-based (“situated”) learning over time, with more direct scaffolding and guidance by PD tools and PD leaders at the beginning of the program that moves toward more teacher-directed work at the end of the 1-year program. The cognitive apprenticeship phases of modeling, coaching and scaffolding, and fading guide how the PD leaders work with participants across time.

## Implementation design principles for STeLLA video-based analysis of practice

Because this special issue focuses specifically on video-based PD, we will anchor our description of the implementation of the STeLLA PD around a central activity in the program: video analysis-of-practice (design principle 5). In this section, we describe how the video analysis work is supported and linked to other design principles, highlighting ways in which the design principles work together to support meaningful video analysis.

Although we are highlighting the role of video analysis in the STeLLA program, it is important to keep in mind that teachers in this program are doing more than video analysis. Their analysis-of-practice work (design principle 5) includes analysis of student work, especially the comparison of students’ pre- and posttest writing, and the analysis of the STeLLA lesson plans. In addition, teachers are engaged in teaching STeLLA exemplar lesson plans in the fall and in planning and teaching lessons in a second content area in the spring (design principle 7). Teachers have opportunities to learn from this planning and teaching work that go beyond what they are learning from video analysis work.

### Design principle 5: video-based analysis of practice

What is being analyzed in STeLLA video analysis? Before describing what teachers and PD leaders do in STeLLA video analysis, we start by linking back to the foundational design principles 1–3 and the importance of clearly defining the substance of the PD program; that is, what are teachers going to learn from this video analysis work? What are they analyzing and why?

While most descriptions of PD programs in research reports provide only a broad, general view of the program substance, we attempted to be more specific in defining what teachers would learn from video analysis while at the same time resisting the temptation to “cover” everything that we know is important in achieving the best practice in science teaching. For the science content, we identified science ideas in the teachers’ curriculum that we knew to be challenging for students and teachers, to be useful in explaining a variety of phenomena in teachers’ and students’ experiences, and to be linked to important crosscutting concepts (e.g., matter and energy).

To identify the STeLLA teaching strategies, we reviewed the research on science teaching and learning to identify what others now refer to as “high-leverage teaching practices” (Ball and Forzani 2011; Windschitl, Thompson, Braaten, and Stroupe 2012: Grossman, Hammerness, and McDonald 2009). And it was important to us that the lenses and strategies in the conceptual framework were not just a random collection of teaching strategies but that they hung together to help teachers develop a vision of effective science teaching that they could internalize over time. Thus, before we decided how to organize and structure video analysis, we worked hard to clarify why teachers would be analyzing videos and to sharpen our understanding of what they would be learning from this work.

What is the form of STeLLA video analysis? Descriptions of PD programs often focus more on the form of the program than on the substance—how teachers are organized to work together, how often they meet, when and where they meet, for how long, who leads the work, the kind of activities they engage in, and so forth (Kennedy 1999). The following features of program form are included in the version of the consensus model presented earlier: active participation of teachers in analysis of practice, sufficient duration, and collective participation of teachers (teachers at a given school or grade level). The STeLLA design principles include attention to each of these consensus model features of program form. Principle 5 defines analysis of practice as a core active learning activity for teachers. Teachers are also engaged actively in content deepening activities (principle 6) and teaching activities (principle 7). Principle 8 identifies program duration and intensity as a key feature, with STeLLA teachers meeting together for 2 weeks in the summer followed by monthly ½ day meetings during the school year. In between meetings, they teach STeLLA lesson plans and collect and analyze student work. Principle 9 addresses the collective participation of teachers at the same grade level. STeLLA teachers meet face-to-face in small grade-level specific study groups (5–8) that are led by experienced PD providers.

But there is an important way in which the STeLLA program goes beyond the consensus model in defining its program form. Decisions about STeLLA program form are driven by the situated cognition of teacher learning and the cognitive apprenticeship model of instruction (foundational design principle 4). This is especially evident in the way different kinds of activities are sequenced over time.

In the summer institute, teachers begin the analysis process using videocases from more experienced teachers. In addition to the modeling provided by these teachers in the videos, PD leaders model how to productively analyze these videos and they coach teachers in their analytical efforts. Thus, in line with our theoretical stance, teacher participants have access at the beginning of the program to models of expertise.

During the fall of the school year, they transition to analyzing video their own and their peers’ efforts to teach STeLLA model lesson plans, receiving support, feedback, and guidance in this work through their collaborative analyses of videos and student work in study group interactions. This is consistent with the coaching phase of the cognitive apprenticeship model.

In the second half of the school year, scaffolding supports such as model lesson plans are removed (fading) and teachers are challenged to use STeLLA strategies and tools to plan and teach lessons in a new content area. This intentional use of a theory of teacher learning required us to consider what types of activities would be most beneficial for teacher learning across time, rather than settling on one type of video analysis and using it throughout the program.

What features contribute to productive video analysis? While the foundational design principles drive the substance and form of STeLLA video analysis, there are a number of other features that work together to make important contributions to the quality of video analysis and to teacher learning:

#### Design principle 10: shared science content

In line with our situated cognition theory of teacher learning, it is essential that teachers work on science content that is in their grade-level curriculum. This assures that each teacher is working in a meaningful, authentic context. In addition, when teachers are all using the same content learning goals, it enables shared experiences that allow for deeper analysis of practice and for development of common understandings of the content and teaching strategies.

#### Design principle 11: educative curriculum materials and assessments

In the fall, teachers are supported in teaching exemplar lesson plans that are designed to highlight anticipated student thinking, the science content storyline, and the STeLLA strategies. These educative curriculum materials are intended to scaffold teachers’ developing understanding of the science content and their beginning use of the STeLLA teaching strategies. For each content area, there is a set of 6–12 lesson plans and a pre-post assessment. As with design principle 10, these shared curriculum materials provide participants with a common teaching experience that enables deeper and more meaningful analyses of that practice.

#### Design principle 12: both exemplar and participant videos

In the STeLLA program, teachers begin the video analysis process in the summer by analyzing the practice of other teachers shown in grade-level and content-specific videocases prepared by the STeLLA program development team. These videocases include classroom video of science teaching that shows the STeLLA strategies in action, video of student interviews about the science content before and after an instructional sequence of lessons, and written student work on pre- and posttests. The STeLLA researchers review these videocases and select clips that will be used during the summer institute to engage teachers in developing their initial understandings of (a) the science content, (b) the STeLLA teaching strategies, and (c) the video analysis process. During the school year, teachers use the STeLLA lesson plans and are each videotaped during one lesson. During study group sessions, teachers analyze video clips of the implementation of these lessons from their own and their colleagues’ classrooms.

#### Design principle 13: analysis tools and processes

Analytical tools and processes support teachers in deepening their analysis of science teaching and learning. These include (a) video clip transcripts which are essential for evidence-based reasoning, (b) analysis guides to support teachers in assessing the quality of implementation of each Science Content Storyline teaching strategy, (c) a STeLLA Lesson Analysis Protocol which guides video analysis, (d) a features analysis chart that reveals patterns in student written work, and (e) lesson planning tools that support teachers in developing student-focused lessons with coherent science content storylines.

The Lesson Analysis Protocol (LAP) is the core analytical tool that is used throughout the program. The LAP process involves three main steps: (1) identify, (2) analyze, and (3) reflect. As shown in the example of an LAP in Table 3, participants first watch the video to identify instances of the use of selected strategy(ies) stipulated on the protocol. The goal here is to deepen teachers’ understanding of the targeted STeLLA strategies. The analysis phase is structured around one or more analysis questions that the PD leader has created and entered on the LAP sheet (see Table 3). Teachers revisit the video and/or the transcript and write an analysis in response to one of the analysis questions. Four components are required in these analyses: (1) a claim related to one of the analysis questions, (2) evidence from the video or transcript to support the claim, (3) reasoning from STeLLA resources and/or research that clarifies why the claim and evidence are important, and (4) an alternative interpretation, suggested teaching approach, or a missed opportunity. During discussion, teachers share and discuss their analyses. The process ends with participants reflecting on what they learned from the analysis discussion.

**Table 3:** STeLLA example lesson analysis protocol

STeLLA Lesson Analysis Protocol: Teacher Name		
1. Identify the Lens & Strategy • What instances of asking questions that elicit, probe, and challenge student thinking do you observe? • What instances of engaging students in interpreting and reasoning stout data and evidence do you observe?		
2. Analyze the Video Using the Focus Question(s) • What do students seem to understand (or not) about the sun’s effect on climate and seasons? • In what ways did the teacher engage students in interpreting and reasoning about data and observations? How did the use of the strategy make student thinking more visible?		
Lesson Analysis Step	To Do	Your Analysis
Claim	Turn an observation, question or judgment into a specific claim that responds to the focus question.	
Evidence and reasoning	Point to a specific place in the video transcript lesson plan, or student work that supports your claim. Connect your claim and evidence with reasoning based on STeLLA Strategies, research on learning, your teaching experience, or scientific principles. Also look for evidence that challenges your claim. Consider an alternative interpretation or explanation.	
Alternatives	Consider new questions this might raise. Consider alternative question’s), activity(s), or strategies.	
3. Reflect What did you learn from this lesson analysis experience?		

#### Design principle 14: reference materials

There are two key reference documents that are used extensively at the beginning of the program and are then referred back to throughout the program to support teachers in developing and maintaining common understandings of the STeLLA conceptual framework and strategies and of the science content. In the summer institute, teachers first learn about each of the STeLLA strategies by reading about them in the *STeLLA Strategies* booklet. During the school year, this booklet is frequently used as a resource to support teachers’ claims, evidence, reasoning, and alternatives in the lesson analysis process.

Teachers also refer often to a topic-specific content and pedagogical content background document. On the one hand, this is a content background document that is matched to the student and teacher science learning goals in the STeLLA program. But it is also a pedagogical content knowledge (PCK) document. In this regard, it describes the science content in the context of teaching situations, analyzes common student misunderstandings and difficulties, and describes strengths and weaknesses of common teaching activities, analogies, and content representations.

## Leadership design principles for STeLLA video-based analysis of practice

### Design principle 15: PD leadership

In the STeLLA design, leadership of analysis-of-practice work plays a critical role in deepening teacher learning. Our choice of the title, “PD leaders,” reflects our recognition that leadership is necessary for the kinds of transformative change we seek. Some might view this as too “top down” and argue for an approach where teachers and PD leaders are co-constructing knowledge together (this is what some refer to as “facilitating” rather than “leading”). But our experience early on in the development of the STeLLA program convinced us that elementary teachers—who are typically reluctant to teach science—initially need both access to expertise from the science education community and support in learning to incorporate this knowledge into their science teaching practice. With fading supports from PD leaders over time, study groups gradually begin to function in a more bottom up way. In fact, we can imagine experienced STeLLA study groups functioning productively without a PD leader as they continue beyond the 1-year STeLLA program because they know how to analyze science teaching and learning productively to improve their science teaching practice, they know when and where to find outside expertise, they understand how to function as a true *learning* community, and they understand the kinds of science knowledge they need to develop when they approach new science content areas—knowledge that enables them to explain a variety of phenomena using key science concepts and practices.

### Design principle 16: PD leader knowledge and decision-making abilities

**Fig. 4:**
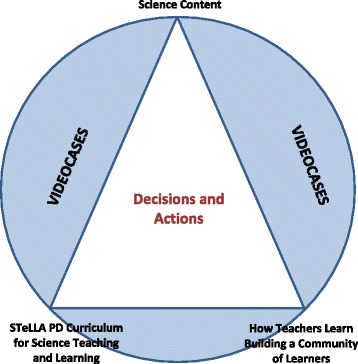
Knowledge and decision-making model for STeLLA PD leadership

It is not enough to say that “PD leadership is important.” In thinking toward the scale up of the STeLLA approach, we recognized the need to understand what it takes to develop new PD leaders: What kinds of knowledge and abilities are needed? Analysis of the work of effective STeLLA leaders enabled us to answer this question with the knowledge and decision-making model of STeLLA PD leadership shown in Fig. 4 (Landes & Roth, 2013).

The model highlights that effective leadership of STeLLA lesson video analysis depends on PD providers who are able to draw from a rich knowledge base to make decisions and take actions—both in planning and while leading video analysis sessions—that support teacher learning through deep and meaningful analyses. The model specifies that well-prepared STeLLA PD leaders make decisions and take actions by drawing from three types of knowledge: (a) knowledge of the STeLLA PD curriculum (conceptual framework, learning goals, STeLLA strategies, STeLLA resources, lesson plans, etc.), (b) science content knowledge (including how science practices can be used to explain phenomena and to understand disciplinary core ideas and cross cutting concepts), and (c) research-based knowledge about how teachers learn in communities of learners. In addition, PD leaders draw from knowledge of the particular videos that are being analyzed. PD leaders know how to use all of this knowledge to make planning and in-the-moment, program-aligned decisions and adaptations.

A key planning decision made by PD leaders is the selection and sequencing of video clips so that they best support targeted teacher learning goals for a given study group session. PD leaders watch the full lesson videos from the participating teachers to select a 3–8-min video clip from each teacher whose video will be analyzed during a given study group session. In making these selections, the PD leaders consider ways in which the video clips can be used (a) to deepen teachers’ understanding of the STeLLA strategies, (b) to clarify and deepen teachers’ science content understandings, and (c) to analyze interesting examples of student thinking. PD leaders also consider how the set of 3–4 video clips for a given study group session can hang together to focus on specific teacher learning goals for the overall meeting. From watching the full lesson videos, the PD leader learns a great deal about the strengths and weaknesses of the lessons and of teacher implementation of the lessons and uses this knowledge to identify key issues that could be productively examined through video analysis. Last but not least, PD leaders consider how a video clip might support and encourage the teacher featured in the clip while also challenging that teacher to reflect on her/his own teaching in ways that lead to growth; this requires assessing that teacher’s readiness to be challenged. All of this is considered as PD leaders select the video clips, identify specific teacher learning goals for the session, and create specific identify and analysis questions to focus the discussion and analysis for each clip.

During video analysis discussions, PD leaders make in-the-moment decisions about when and how to take the following kinds of actions:

- Asking elicit and probe questions to encourage teachers to share and elaborate their thinking
- Asking challenge questions to scaffold and deepen teachers’ analyses and understandings of both science content and pedagogical content (e.g., What is your evidence? Can you find something in the strategies booklet or science content background document that supports your reasoning?)
- Modeling the video analysis process by sharing her/his own claim, evidence, reasoning, and alternative about a video clip
- Modeling and reflecting metacognitively on the PD leader’s use of STeLLA teaching strategies (e.g., PD leaders explicitly name or ask about STeLLA strategies that they are modeling)
- Encouraging participation by all (e.g., directing teachers to do a turn and talk before a whole group discussion, using a round robin strategy to hear ideas from each person, asking questions such “What do others think?,” encouraging teachers to respond to each other and to ask probe and challenge questions to each other)
- Interrupting the video analysis process to engage in some needed science content deepening work
- Highlighting and summarizing key points (e.g., charting key science ideas, summarizing the key ideas coming out of analysis of a video clip)
- Asking teachers to summarize or paraphrase what they have heard
- Providing feedback about the quality and accuracy of teacher ideas (generally limited to times when discussions hit a roadblock and the PD leader assesses that it is not a good use of time to resolve the issue by sending teachers to resources or engaging in a content deepening activity)
- Remaining silent (e.g., letting the conversation be more teacher directed)

A key decision PD leaders make is about when and how to address gaps in teachers’ science understandings that arise during video analysis. Taking the time in video analysis sessions to wrestle with science content confusions is a critical aspect of the STeLLA program. Our research results show that teachers’ science understandings were better developed in the context of lesson video analysis (in the STeLLA program) than in sessions that focused specifically on deepening teachers’ understandings of the science content (in the content deepening comparison program). This is consistent with our situated theory of teacher learning—understanding the science content is more meaningful to teachers when it occurs in relationship to their teaching context.

Without the PD leaders’ active role in scaffolding the video analysis process, especially at the beginning of the program, teachers tend toward a surface level discussion of video clips, favoring positive comments about the teaching or teacher featured in the clip. With PD leader guidance and use of the Lesson Analysis Protocol, teachers’ attention is focused on student thinking, the science content, the storyline, and the use of STeLLA strategies, minimizing the focus on an individual teacher while maximizing the analysis of more consequential issues that impact student learning.

## Design principles for scaling and sustaining the STeLLA video-based PD program

The effectiveness of the STeLLA VbPD program has been demonstrated through careful research—effective not only for teacher learning and practice but more importantly for student learning. But video-based analysis of practice is intense, requiring significant human and other resources to support its success. It is definitely not a quick fix. STeLLA-I and STeLLA-II VbPD programs were designed to be workable and sustainable as long as there was significant project funding from the National Science Foundation. The video focus, the need for transcripts to support evidence-based video analysis, the need for science hands-on materials to support the teaching of the STeLLA lessons, and the facilitated, small study group face-to-face structure of this PD approach pose challenges for scalability and sustainability. The RESPeCT project is addressing the challenge of scaling and sustaining this VbPD approach by (a) developing a cadre of teacher leaders at both the district level (teacher specialists) and school levels (classroom teachers), (b) developing university science and mathematics faculty who can support this work beyond the life of the grant in PUSD or in new school districts, and (c) implementing changes within the district and university systems that will enable teachers and professors to participate effectively in this program.

In this section, we describe more specifically what we are doing in the RESPeCT project to support the scaling and sustainability of this VbPD program. Three design principles guide this work: principle 17 pertains to the development of new VbPD leaders while principles 18 and 19 focus on development and sustainability of systems in which VbPD PD leaders can effectively work.

### Design principle 17: PD leader development

The development of STeLLA VbPD leaders who have the knowledge and abilities described in Fig. 4 is critical to enabling more teachers and students to have access to this powerful program. To address this need, a leadership development program was designed, developed, and enacted in the RESPeCT project. The leadership development program attends to the full scope of the STeLLA knowledge and decision-making model (Fig. 4).

The content of the leadership program and the process of developing VbPD leaders changes based on the needs of the audience. We have worked with a variety of audiences, including (but not limited to):

- Experienced science education PD leaders who are unfamiliar with the STeLLA PD program
- RESPeCT project elementary teachers who have experienced the STeLLA program as learners but have limited knowledge and experience in leading adult learning
- District level PD specialists who have experienced the STeLLA program as learners
- University science and mathematics faculty unfamiliar with the STeLLA approach and with varying levels of experience in working with teachers.

When the audience is experienced science PD leaders with little experience with the STeLLA VbPD program, the balance of leadership development tips toward the content of the STeLLA VbPD curriculum (lower left hand corner in Fig. 4). However, when the audience has experienced the STeLLA VbPD program and implemented the STeLLA classroom curriculum, the balance of leadership development tips toward developing understandings about and abilities to lead adult learning (lower right hand corner in Fig. 4). For science and mathematics professors unfamiliar with either the STeLLA approach or working with teachers, leadership development focuses on the bottom two corners of the triangle in Fig. 4, developing their understanding of the STeLLA VbPD program goals and components and their knowledge about teacher learning in learning communities.

Regardless of audience, one element of VbPD leadership development remains constant—a focus on making decisions and taking actions consistent with the STeLLA approach. Effective PD leaders know the purpose for each component of the program. They understand what they are doing as PD leaders and why they are doing it. Major goals and activities in the leadership program are described in the following paragraphs.

#### Taking on a leadership identity

To help classroom teachers who have not previously led PD see themselves as leaders, we are explicit about how the leadership development program helps prepare them to lead the learning of their teacher colleagues. For each PD experience, we point to a model similar to the figure presented in the introduction to this special issue (Tekkumru-Kisa and Stein 2017, Fig. 5). This model helps RESPeCT teacher participants see where they fit in terms of their experiences as a learner in the STeLLA program (teacher as learner, inner layer), their experiences in the RESPeCT leadership development program (facilitator as learner, outer layer), and their eventual role as PD facilitators (middle layer).

**Fig. 5:**
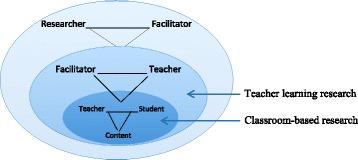
Layers of professional development design (Tekkumru-Kisa and Stein, 2017)

#### STeLLA strategies become PD leader strategies

The leadership development program supports PD leaders in learning to use the STeLLA lenses and strategies in the PD setting. Most of the STeLLA strategies are just as useful in the VbPD context as they are in supporting elementary students’ science learning. PD leaders are supported in learning how to use the STeLLA strategies through three types of analysis experiences: (1) analysis of their own experiences observing PD leaders in the leadership development program, (2) video analysis of other PD leaders working with study groups, and (3) video analysis of their own PD leadership practice after they begin to lead groups of teachers in this VbPD.

#### The balance between supporting and challenging teachers

PD leaders need to find the right balance between “supporting” and “challenging” teachers. By supporting, we mean using PD leader moves that encourage teachers by signaling that their ideas are valued and that they are making progress toward program goals. By challenging, we mean PD leader moves that press teachers to reconsider, revise, or better support their ideas. This often comes with some level of discomfort. PD leaders learn to make decisions about when and how to support or challenge teachers by analyzing video of other PD leaders and later by analyzing video of their own leadership practice, focusing on PD leader moves that support and/or challenge teachers. In working toward this balance, PD leaders learn to be intentional when they pose questions that elicit or probe teachers’ ideas, when they ask questions that challenge teacher thinking, or when they use any of the other STeLLA strategies in the context of teaching professionals.

#### Communities of learners

The leadership development program explicitly addresses how to develop communities of learners. For example, PD leaders consider their own experiences in light of readings about characteristics of professional learning communities (PLCs) (Garmston and Wellman 2009; Hall and Hord 2015; Loucks-Horsley, Stiles, Mundry, Love, and Hewson 2010) and identify aspects of the STeLLA VbPD program that contribute to the development of such communities. Use of the STeLLA study group norms and the Lesson Analysis Protocol for video analysis help to develop shared values and behaviors that provide a safe environment for developing relational trust. Participants come to understand how STeLLA video analysis promotes reflective dialogue and deprivatized practice. Taking a step back, they come to understand how the coherence of the STeLLA lessons, content deepening, and lesson analysis planning work contribute to a collaboration focused on student learning.

#### Understanding change

For audiences with little experience in leading PD, such as RESPeCT teacher leaders or many science professors, the leadership development program includes a focus on leading adult learning. Specific attention is paid to understanding change and what it means to lead change, anticipating and mediating sources of resistance, and managing their own responses during conflict (Hall and Hord 2015). For new teacher leaders in the RESPeCT program, this is particularly important as they anticipate work with their grade-level teacher peers.

#### Selecting video and planning coherent PD study group sessions

Participants in the leadership development program examine models of video clips and rationales for their selection. PD leaders learn to select clips which represent a progression in the use of STeLLA strategies and a storyline of the science content. For example, the first clip in a study group session might focus on an early lesson in the sequence that reveals common student ideas about steam through the use of elicit and probe questions (STeLLA teaching strategies 1 and 2). The second clip may show a later lesson where students negotiate this idea through an activity matched to the learning goal (STeLLA teaching strategy C) in which students analyze and interpret data from their observations of a tea kettle (STeLLA teaching strategy 4). A third video clip may come from later yet in the lesson sequence and show students attempting to use and apply science ideas (STeLLA teaching strategy 6) about evaporation and condensation as they compare/contrast cloud formation and steam from a tea kettle.

#### Deepening PD leaders’ science content knowledge

To effectively facilitate science content deepening, PD leaders must achieve a level of confidence with the content that enables them to identify gaps in participants’ content knowledge and to decide “in-the-moment” how to address them. In the leadership program, content deepening is embedded in two contexts. First, as in the STeLLA program itself, content deepening in the leadership program is addressed by engaging participants (teacher leaders as learners) in video analysis. For example, teacher leaders watch a science lesson clip followed by a study group clip in which teachers are discussing the lesson clip. In the clips, both student and teacher misunderstandings of science content are revealed. This provides an opportunity for teacher leaders in the leadership program to clarify their own content understandings as they also think about how to lead sessions where such misunderstandings arise. Content deepening also occurs as teacher leaders are supported by science and mathematics faculty in practicing their implementation of content deepening segments of the VbPD program.

#### Deepening pedagogical content knowledge

Video analysis is also used to deepen leaders’ pedagogical content knowledge regarding the use of STeLLA lenses and strategies. As participants in the leadership program examine video of study groups, they analyze PD leader knowledge, moves, and decision-making. In this context, opportunities also arise to clarify teacher leaders’ understanding of common student ideas and effective use of the STeLLA strategies related to the target science content learning goals.

#### Practice leading STeLLA PD

The leadership development program engages PD leaders in practice facilitation of both lesson analysis experiences and content deepening activities. To create safe learning contexts, the practice facilitation involves participants in leading content deepening experiences within their own study group and in leading analysis of video to RESPeCT teacher leaders from other grade level study groups. These practice opportunities are scaffolded to include planning, doing, and reflecting stages. PD leader guides support the practice facilitation, providing details about how to implement each summer institute and school year study group session. These guides are linked to associated PowerPoints, handouts, and video clips.

With the development of new PD leaders across the STeLLA-II and RESPeCT implementations, the STeLLA VbPD program has expanded its reach by developing 50 new PD leaders who in turn have worked with 200 teachers and their 7019 students. Through this multi-stage process, we learned that you cannot shortchange any component of the STeLLA model of PD leader knowledge and decision-making. An effective STeLLA PD leader understands the science content, the many facets of the STeLLA PD and classroom curricula, how teachers learn and change their practice in a community of learners, and how to use this knowledge to lead productive video analysis.

### Design principle 18: partnership development and design principle 19: scalability and sustainability

While the STeLLA VbPD approach has demonstrated strong results in terms of teacher and student learning, it is an ambitious program that faces challenges in expanding the reach of the program. One challenge is the development of new, grade-level specific PD leaders who have the knowledge and abilities described in the previous section. Other requirements that might be impediments to long-term sustainability are the need for funding and other benefits to motivate teacher PD leaders in continuing to offer the VbPD to peer teachers; funding and other rewards to encourage teachers to participate in the VbPD; support and strategies for filming classrooms and making, transcribing, and disseminating video clips on a tight timeline; time for teacher PD leaders to review lesson videos and plan study group sessions; funding for and distribution of PD and science lesson plan materials; stable student science learning goals at each grade level so that the videocases remain relevant; stable grade-level teaching assignments so that teachers are teaching the content addressed in the VbPD program; supports to motivate and sustain university faculty commitment to providing ongoing science content advice to teacher PD leaders beyond the life of the grant; refilling the pipeline of teacher leaders and university support faculty; and strong district and university leaders who can lead successful responses to changes in staffing, curriculum, and institutional priorities that impact the VbPD program.

To preserve fidelity to the STeLLA design features and sustain the VbPD program, we must do more than prepare new teacher PD leaders. Educational systems must also be readied. In the RESPeCT project, the systems involved in sustaining the work are the Pomona Unified School District and the California State Polytechnic University at Pomona.

Together, but not individually, CPP and PUSD have the needed expertise identified in design principle 18: science education experts and researchers, scientists and mathematicians, teachers, and school personnel at multiple levels. This combined expertise is necessary to modify the VbPD program in ways that preserve the STeLLA design features while incorporating features that will contribute to the program’s scalability and sustainability. Thus, neither organization can scale and sustain the program independently of each other. However, the components of readiness required by both partner systems include (a) a shared priority for improving student science learning, (b) shared values and commitments to VbPD; (c) ongoing communication and shared decision making to support, sustain, and study the collaboration; (d) investments in building capacity for VbPD; and (e) inclusion of multiple stakeholders in shaping and implementing the program.

### Developing systems readiness

Both CPP and PUSD partners have worked on developing these components of system readiness, as described in the following paragraphs.

#### Priority for improving student science learning

In response to national and local testing and funding policies, many districts and schools have raised instructional time requirements for elementary English language arts and mathematics which have had the effect of largely squeezing science out of the curriculum (Dorph et al. 2011). This is not the policy of the PUSD central offices, who are particularly interested in improving elementary science teaching in light of the impending curriculum shift to match the NGSS and the testing of science at the fifth grade level in California. However, in practice, many PUSD elementary teachers put a low priority on science teaching. During the writing of the RESPeCT grant proposal, the PUSD and CPP partners agreed on the importance of making science learning a higher priority in the district. Project leaders agreed upon ways to modify the STeLLA program to provide more incentive for teachers to prioritize science teaching. One modification was to integrate key Common Core English language arts and mathematics standards into the science lessons that are the focus of the videocases in the VbPD program. In this way, teachers can see science instructional time as also time for developing important literacy and math competencies.

#### Shared values and commitments to VbPD

In addition to the shared commitment to improving science learning in the district, the project partners from the beginning held a shared vision about and a commitment to the goals and design of VbPD work. This shared vision was developed through four main joint activities: previous partnership work in PUSD middle schools that included some video analysis work using the STeLLA conceptual framework, study of the published research about the STeLLA program, collaborative writing of the RESPeCT proposal, and the enactment of the VbPD program over the past 3 years. This shared commitment to VbPD helped build a mutual respect for the different types of knowledge, expertise, and credibility that the university and the district bring to the work; a commitment to acknowledge and balance each institution’s priorities and constraints to ensure the partnership is mutually beneficial; and active support of the program and flexibility about what can be achieved together.

In terms of flexibility, both partners are committed to exploring better ways to do the work and to find creative solutions to obstacles, no matter how big or small. Both organizations deal with changes that are difficult for the partners and partnerships. For example, PUSD principals and central administrators made a commitment to keep RESPeCT teacher leaders at the same grade level at least for the life of the 5-year grant. When circumstances arose that made this commitment difficult to keep, administrators consulted with CPP researchers to find a compromise that would satisfy project needs.

#### Communication and shared decision-making

Strong partnerships are maintained by communication which occurs often, is clear, and uses formative data to make mid-course adjustments. Regular meetings with the various stakeholders are important for clarifying roles and responsibilities of each institution. In the RESPeCT program, groups that meet regularly include the Principal Investigator Leadership team (which includes CPP faculty and the PUSD deputy superintendent), the evaluation team, the research team, the university science and math faculty team, the supporting partner team at BSCS, the treatment schools principal team, the district support staff team, the university support staff team, and the university student support team. To assure these groups are working in concert with each other, there are regular cross-team meetings such as monthly PI/evaluation team meetings. Meeting time provides space for updates and problem solving, while also creating space for the various partners to contribute to decisions and to co-construct the ongoing collaborative work.

While we have strong norms for communication and shared decision-making, the process of developing these has not been perfect, in part because of different cultures around decision-making and authority. For example, university faculty are accustomed to having a great deal of freedom in making their own decisions about their professional activities and outside-of-class schedules. For some, the RESPeCT research project involves more people and more collaboration than their science research projects and provides them with less authority to make decisions about their roles and timelines for project work. It took careful two-way communications, mentoring, reasonable modifications of expectations and timelines, and writing templates and leadership guides to keep faculty involved and supportive of project goals and activities.

#### Investments in capacity building

To sustain the PD leaders and the partnership, thoughtful investments in capacity are critical. Collaborative work requires human capital, funding, and time. The RESPeCT partners work together to ensure that funds support the recruitment of and dedicated time for staff, faculty, and PD leaders with the expertise needed to implement the VbPD program; the recruitment of and support for teachers and district teacher specialists to participate in the PD; and the classroom materials, technology, and physical space needed to support the PD program and the teaching of the science lessons. An additional element of human capital we cannot overemphasize is the importance of having a strong support staff who can make sure that all parts of the program are coordinated, communicated, and scheduled appropriately. In addition to the usual roles played by support staff, the following needs of a video-based program that is also part of a research study are addressed: scheduling the videotaping of science lessons; training student videographers; videotaping science lessons and study group sessions; making video clips; producing transcripts of video clips; disseminating videos and video clips; and organizing systems for filing and sharing videos, video clips, and supporting materials.

From the beginning of the RESPeCT project, the leadership team engaged in strategic planning and capacity building to ensure that the VbPD program can be supported to reach all K-6 teachers in the district after grant funds expire. The post-grant phase will necessitate difficult conversations and compromises, largely related to funding, but we have laid a strong foundation for a collaborative and reflective professional community within and across institutions that includes patience, compromise, and flexibility in problem solving.

#### Involving all stakeholders

In order to change the PUSD and CPP systems in ways that will sustain the RESPeCT program, the partnership institutions must move beyond the interactions of the leadership team to engage administrators, faculty, staff, and students at both the university and the district. Toward this goal, the PI team creates opportunities for a wide range of stakeholders to take authentic roles in collaborative activities. The RESPeCT program directly includes collaborative roles in the PUSD district for 35 teacher PD leaders, six treatment school principals, four teacher PD specialists (English Learner, English Language Arts, Primary Education, and Secondary Science), district level support staff, the Director of District Professional Development, the Director of Principal Support, and the Deputy Superintendent. At the university, science and mathematics content faculty, education faculty, a science content department chair, the Educational Outreach Center department chair, and CPP undergraduate students all play collaborative roles in the project. Engaging a diverse set of stakeholders is imperative for ensuring that the program meets the needs and interests of those who stand to benefit, namely the elementary teachers and students, but also to sustain the program despite turnover among critical individuals.

In addition to casting a broad net for stakeholder involvement, key individual leaders at both the district, school, and university levels play critical roles in maintaining everyone’s confidence in the program’s organization and in keeping teachers fully participating in the program. These key champions play a “boundary-spanning” or “bridging” role (Bosma el al. 2010; Goldring and Sims 2005; Weerts and Sandmann 2008) to ensure communication across institutional settings, advocate for program support across the many institutional stakeholders, bring awareness of partnership goals and activities, and invite stakeholders to learn about and provide feedback on partnership goals and activities.

### Importance of research on student learning

Improving student learning outcomes is essential to sustain the current high level of support for the RESPeCT project. Both partners initially bought into the program largely based on the multiple and strong lines of evidence of effectiveness of this VbPD approach not only for teacher learning and teaching practice but also more importantly for student learning. Thus, high-quality research is essential for sustaining this work.

Strong preliminary analyses of teacher and student science learning and the enthusiasm of participating teachers are fueling efforts in the district to find ways to fund this work in the future. For example, PUSD is already committed to supporting ongoing RESPeCT PD work by using the district’s summer PD time for the RESPeCT program, taking on the tasks of purchasing and supplying science kits, scheduling the videotaping of science lessons, creating video clips and transcripts of those clips, and copying and organizing binders for future teacher participants. In support of these district commitments, the RESPeCT leadership team and CPP support staff are experimenting with ways to make the videotaping, uploading and sharing of video, clip making, and transcription processes easier for teacher leaders and Pomona support staff to do. In addition, some of the CPP science faculty are interested in continuing to provide district RESPeCT teacher leaders with “phone a friend” science content support even after the end of the grant.

Impact on student learning is also important at the university. In addition to formal research goals, the project also hopes to improve science and mathematics teaching and learning at the university through participating professors’ implementation of STeLLA strategies in their university classrooms. In addition, we anticipated from the beginning that some CPP science and math faculty will become ready and motivated to start up and support RESPeCT PD efforts in new school districts, especially those in areas surrounding the university so that future CPP students receive a strong foundation in their scientific understandings. Toward this goal, the project currently supports CPP science and math faculty with mentoring, modest reassigned time, experiences that help them learn about the K-6 teaching context in needy Title I schools, and involvement in education research and publication. Continued university support is needed to encourage faculty to engage in VbPD work beyond the life of the grant.

## Discussion and conclusions

There is wide interest in video-based, analysis-of-practice PD as an effective approach to preparing teachers to meet the expectations of the *Next Generation Science Standards* in the USA and in STEM education reform agendas in other countries. But few studies actually examine impact of such PD on student learning. The STeLLA line of research is a rare exception. This special issue of *The International Journal of STEM Education* gave us the opportunity to share what is going on behind the scenes of this effective program, showing how design principles shaped the development, implementation, leadership, and scale up of this particular VbPD program. In the body of this paper, we described the design principles. We now consider the implications of this work for others involved in video-based professional development work: What do the STeLLA design principles contribute to the existing knowledge base about effective video-based, analysis-of-practice PD?

### The power of the set of STeLLA design principles

We argue that this set of design principles is powerful and useful beyond this one project for four reasons. First and foremost, the use of these design principles produced a video-based, analysis-of-practice PD program that had a significant impact on teacher learning, on teaching practice, and, most importantly, on student learning. In addition, teaching practice was demonstrated to be highly correlated with student achievement. Second, the design principles were built on existing theoretical and empirical research literatures. Third, none of the design principles were dropped or significantly revised as the program moved over a 10-year time span to different geographic locations, school district contexts, and grade levels. In fact, the design principles proved to be critical guides in determining how changes would be made to adapt to new contexts without losing the power of the program’s impact on teaching and learning. For example, as the program moved in 2009 from STeLLA-I in California to STeLLA-II in Colorado, the design principles played a key role in guiding our work with new state science teaching standards and in supporting us in resisting the temptation to tailor the program to the specific wishes of each school district. Adaptations were made only when they were consistent with the design principles. Similarly, we responded to the release of the NGSS in 2013 by reviewing our conceptual framework in light of this reform document. We were able to make minor changes to the STeLLA teaching strategies and the language describing these in the STeLLA strategies booklet without needing to change our overall conceptual framework. Thus, the design principles remained largely stable over time, another indicator of their power.

Finally, the design principles have power because they are closely interwoven and supportive of each other. For this reason, it was difficult to write about them separately in this paper. For example, productive learning through video-based, analysis-of-practice (principle 5) is dependent on working in a context where teachers feel safe to work with their colleagues as they to dig deeply and challenge themselves and each other (principle 9). Such challenges and deeper analyses are more likely when tackling shared content- and grade-level specific curriculum (principles 2, 3, 10) and using the same curriculum materials (principle 11). Feeling safe in a learning community is not productive if you are not challenged to stretch and change your thinking. In the STeLLA line of research, we have found that such challenges can come from knowledgeable and skilled PD leaders (principles 15, 16), as well as from models by more experienced STeLLA teachers (principle 12). But the ultimate goal is to create learning communities where such challenge can come from teacher participants themselves (principle 9) as they learn from videos from their own classrooms (principle 12). And all of this takes time (principle 8).

### Challenging the consensus model of effective PD

At the beginning of this paper, we made the case that the long-accepted and widely used consensus model of effective PD is of limited value in guiding the development, implementation, leadership, and scaling up of VbPD programs that positively impact teaching and student learning. We argued that this model lacks research support showing that it is effective in improving teaching and student learning. In addition, it provides only broad, surface level recommendations about how to design and implement PD experiences: Effective PD is content-focused, engages teachers in active learning, is matched to local, state, national policies, is of sufficient duration, and involves collective participation of teachers at a school or grade level.

The STeLLA VbPD program supports the consensus model with strong empirical evidence of student learning. But the STeLLA model also advances and deepens our understandings of effective PD by digging beneath the surface of the consensus model to articulate design principles that provide more specific guidance about features of effective PD. And, importantly, this model is supported by strong research evidence of impact on teaching practice and student learning. Based on our experiences with STeLLA, we nominate the following design principles as candidates that might be added to the consensus model and further tested for their potential as design principles to guide all VbPD:

- A clearly articulated conceptual framework that guides the substance of the program (principle 1)
- Clearly specified learning goals for teachers (science learning goals, PCK, and teaching practice) and students (science learning goals) (principle 2)
- Program substance that prioritizes depth over breadth (principle 3)
- A theory of teacher learning to guide the structure, organization, and form of the program (principle 4)
- Video-based analysis-of-practice guided by an explicit analysis process (principles 5, 11)
- Shared content, curriculum, and curriculum materials (e.g., grade level specific, teaching the same lessons) (principles 10, 11)
- Educative curriculum materials (principle 11)
- Science content learning experiences that are intertwined with and grow out of analysis-of-practice work (principle 6)
- Scaffolded teaching practice (principle 7)
- Expertise and leadership support from PD Leaders and clear specification of how their roles change over time (principles 15, 16)
- Support for PD leader development (principle 17)

Of course, a refined consensus cannot be built based on findings from this one study. Additional research is needed before this list of nominated features can change the field’s consensus about effective PD (and effective VbPD, in particular). There is some existing research that examines the impact of science PD on both teacher and student learning and supports some of these STeLLA nominations. For example, results from the Heller et al. study (2012) support the intertwining of science content learning with analysis-of-practice activities. The importance of scaffolding by knowledgeable PD leaders emerged in studies by Greenleaf et al. (2011), Heller et al. (2012), and Penuel et al. (2011). But none of these studies looked specifically at video-based PD. Clearly more research is needed.

We hope that this analysis of lessons learned from the STeLLA design principles can be useful for the design, implementation, and study of other professional development experiences for teachers, especially those that focus on video-based analysis of practice. We believe that an evidence-based articulation of VbPD design principles for the field would represent a major step forward in our knowledge about effective PD. This could help us move from the current surface level recommendations about “effective” PD that are today so widely used to guide the development of science teacher professional development programs to a set of well-specified, coherent design principles that are linked to evidence of student learning.

## Appendix 1

**Table 4:** Summary of STeLLA Student Thinking Lens strategies

	Strategy	When	Purpose
Questions that reveal and challenge student thinking	1. Ask questions to elicit student ideas and predictions	Before learning goal is developed	To reveal students’ initial ideas, predictions, misconceptions, and experiences
	2. Ask questions to probe student ideas and predictions	Any time	To reveal more about a given student’s current thinking
	3. Ask questions to challenge student thinking	As part of developing the learning goal	To challenge student thinking in the direction of the learning goal; to help change student thinking about the science ideas
Activities that challenge student thinking	4. Engage students in analyzing and interpreting data and observations	As part of developing the learning goal or after a learning goal has been developed (as a “use and apply” activity)	To teach students how to organize, present, and analyze data in ways that will reveal important patterns and relationships that can be used in developing explanations
	5. Engage students in constructing explanations and arguments	As part of developing the learning goal or after a learning goal has been developed (as a “use and apply” activity)	To engage students in using evidence and science ideas to explain observations and data, and to develop arguments to assess the strengths and weaknesses of competing explanations.
	6. Engage students in using and applying new science ideas in a variety of ways and contexts	After learning goal has been developed	To engage students in using newly learned science ideas to explain new situations, new phenomena, and new real-world connections; to demonstrate the wide usefulness and value of the new ideas
	7. Engage students in making connections by synthesizing and summarizing key science ideas	After learning goal has been developed	To engage students in making connections among ideas, evidence, and experiences they have encountered in the lesson(s)
	8. Engage students in communicating in scientific ways	Any time	To engage students productively in science practices and discourse

## Appendix 2

**Table 5:** Summary of STeLLA Science Content Storyline Lens strategies

	Strategy		Purpose
Develop the science content storyline during planning	A. Identify one main learning goal		To identify the complete science concept you want students to learn
	B. Set the purpose with a focus question or goal statement		To provide a focus for the lesson that keeps students’ attention on the main learning goal
	C. Select activities that are matched to the learning goal		To select activities that help students understand the main learning goal
	D. Select content representations and models matched to the learning goal and engage students in their use		To select representations that help students deepen their understanding of the main learning goal
	E. Sequence key science ideas and activities appropriately		To develop a storyline that will make sense to students
	I. Summarize key science ideas		To plan how the storyline will be tied together
	Strategy	When	Purpose
Develop the science content storyline during teaching NOTE: Planning is a critical step in being prepared to revisit, highlight, and link.	B. Set the purpose with a focus question or goal statement	At the beginning and highlight throughout	To focus students’ attention on the purpose of the lesson
	F. Make explicit links between science ideas and activities	□ Before each activity □ During each activity □ After each activity	To make the science content storyline visible to students To engage students in thinking about the science ideas related to the activities
	G. Link science ideas to other science ideas	□ Beginning: Link to ideas from previous lessons □ During lesson: As appropriate □ End: Link ideas developed during the lesson and previous lessons foreshadow next lesson	To make the storyline visible to students To engage students in thinking about the connections among science ideas
	H. Highlight key science ideas and focus question throughout	Multiple times during the lesson	To make the main learning goal and supporting ideas more visible to students
	I. Summarize key science ideas	End of lesson	To tie the storyline together

## Abbreviations

BSCS: Biological Sciences Curriculum Study
CPP: California State Polytechnic University, Pomona
LAP: Lesson Analysis Protocol (STeLLA)
NGSS: Next Generation Science Standards
NSF: National Science Foundation
PCK: Pedagogical content knowledge
PD: Professional development
PI: Principal Investigator
PLC: Professional learning community
PUSD: Pomona Unified School District
RESPeCT: Reinvigorating Elementary Science through a Partnership with California Teachers
STeLLA: Science Teachers Learning from Lesson Analysis
TIMSS: Trends in International Mathematics and Science Study
VbPD: Video-based professional development
